# Educational environment and its relation to academic performance as perceived by medical students in University of Khartoum. A cross Sectional Study, Sudan, 2020

**DOI:** 10.15694/mep.2021.000170.1

**Published:** 2021-06-11

**Authors:** Duha Mohammed, Eilaf Yassen, Mozan Mohamed, Raghad Eltayeb, Rofida Asmally, Sara Mohammed, Sujood Elhassan, Gehad Ibrahim

**Affiliations:** 1 University of Khartoum - Faculty of Medicine; 2 Sudan Medical Specialization Board

**Keywords:** Educational Environment, DREEM, Sudan, Medical School, Medical Education

## Abstract

This article was migrated. The article was marked as recommended.

**Purpose:** to assess students’ perception of educational environment and to evaluate the difference in perception related to academic performance and demographic characteristics in the Faculty of Medicine, University of Khartoum, 2020.

**Method:** The authors performed an observational analytical cross-sectional study at the Faculty of Medicine, University of Khartoum, using a validated structured closed-ended self -administered questionnaire; the Dundee Ready Educational environment measurement (DREEM). They included all students registered in the faculty for the academic year 2020-2021 except the first semester students. They used proportionate stratified sampling to divide students into strata and sub-strata based on batch number and sex, respectively. Then, they selected students proportionately from each substrata using simple random sampling.

**Results:** out of 405 randomly selected students, 341 (84.2%) filled the questionnaire. The mean total DREEM score was (104.48/200), indicating a positive perception of the learning environment. This, with only student’s perception of atmosphere subscale showing negative results. Test results showed a significant difference between different student’s age groups, academic phases and batches in regards to their perception of atmosphere (p-value 0.023, 0.001, 0.013 respectively). Regarding residence, a significant difference was found in total DREEM scores, with students residing in university dormitories having a more positive perception. Test results showed no significant difference in student’s perception of educational environment in all DREEM subscales between achievers and underachievers.

**Conclusion:** although the overall perception of educational environment was more positive than negative, the study highlighted various areas needing special attention. Theauthors believe that both faculty administration and students must work together to deliver tangible improvement in all aspects of educational environment.

## Introduction

For medical students, the term “educational environment” indicates all the physical conditions - including lecture rooms, laboratories, hospital wards, out-patient clinics, curricular contents ( Dunne, McAleer and Roff, 2006)- social and intellectual conditions, forces and external factors represented by teaching staff and their general practice and attitude inside the class in addition to the methods they use to deliver learning materials to students ( Miles *et al.,* 2012). Furthermore, the educational environment for medical students includes their colleagues as well as social and cultural perspectives challenging their performance ( Sharkawy, Houfey and Hassan, 2015). Therefore, the learning environment is an interactive network of forces within the teaching and learning activities that influences students’ learning outcomes and affects the learning process whether directly or indirectly ( Hamdan *et al.,* 2020).

However, despite the fact that the educational environment concept is rather subtle, its effects can’t be neglected ( Ahmed, Zafar and Samad, 2019); hence many authorities including The UK Standing Committee on Postgraduate Education emphasized the importance of educational environment by stating that “A working environment that is conducive to learning is critically important to successful training” ( Salih *et al.,* 2018). It has also been suggested that success of an effectual curriculum along with students’ academic progress strongly relies on the educational environment of a particular medical school ( Naik and Singh, 2017). This in addition to earlier studies affirming that encouraging learning environment; along with a positive institutional profile are major determinants of the students’ desire and motivation to learn; with the greatest impact on increasing satisfaction with the course of study, achievement and success of medical students as well as incentivizing sense of well-being. In contrast, a negative one hinders the progress of students ( Bhosale, 2015; Sharkawy, Houfey and Hassan, 2015; Mushtaq *et al.,* 2017; Walankar *et al.,* 2019). Evidence also exists for the fact that students’ levels of psychological distress are greatly influenced by their learning environment ( Enns *et al.,* 2016).

In that sense, the evaluation of educational environment has been regarded as key to the delivery of high quality medical education. One way to make that possible is the Dundee Ready Education Environment Measure (DREEM), a measure that was introduced in 1997 in Dundee, Scotland, UK through the Delphi Panel; it has been demonstrated to be both a reliable and a valid inventory ( Roff *et al.,* 1997). Recently published data of a systematic review reported DREEM to be the most consistent tool for measurement of the educational environment in the undergraduate medical studies setting ( Miles *et al.,* 2012).

Educational environment evaluation is agreed to have a significant role in managing medical schools. Moreover, continuous reports on students’ perception of their educational environment are encouraged -especially for schools receiving poor fund supports as the situation in Sudanese medical schools - for their remarkable contribution in generating cost-effective interventional plans ( Fahal, 2014). Accordingly, the purpose of our study was to assess students’ perception of educational environment, and the difference in perception related to academic performance and demographic characteristics in the faculty of medicine, University of Khartoum, 2020.

## Methods

### Study design and instruments

We conducted an observational analytical cross-sectional study at the Faculty of Medicine, University of Khartoum, to study medical students’ perception of educational environment, and its relation to academic performance for the year 2020 using the Dundee Ready Educational environment measurement (DREEM). This tool uses a 50-item measure with five scales score (Likert scale 1-5) comprising the following respects of the educational environment: the perception of teaching, perception of teachers, academic self-perception, perception of atmosphere, and social self-perception ( Soliman, 2017).

DREEM is widely employed for the assessment of the educational environment in medical schools. It was designed to develop a non-culturally specific instrument ( Roff, 2005), as reflected by its use in a wide variety of medical schools world over to locate the strengths and weaknesses of educational institutions, to compare the performance and efficacy of various medical schools, and to contrast the perception of the academic environment among students of varying academic years and genders. Moreover, It can also be utilized to guide the process of curriculum modification and to compare it with previous ones ( Miles *et al.,* 2012; Altemani and Merghani, 2017).

### Study population and sample size

Total number of currently enrolled students in the faculty is 2700 students distributed into 6 academic years who progress in their studies from basic to para-clinical and clinical phases ( University of Khartoum, 2008). We included all students registered in this institution for the academic year 2020-2021 except the first semester students (batch 97). We calculated sample size using EPI-INFO version 7, with a 5% *level of precision* and 95% *confidence interval*; knowing that the *population size* is 2363 students we found the sample size to be 330 students. Considering a *nonresponsive rate* of 20%, we estimated the sample size of the study to be 396 students.

We used proportionate stratified sampling. We considered each batch enrolled in the faculty for the academic year 2020-2021 as a stratum. In each stratum, we divided students into two substrata according to their sex (males/females). Then, we selected students proportionately from each substrata using simple random sampling.

### Data collection

We collected data using a validated structured closed-ended self-administered questionnaire; the Dundee Ready Educational environment measurement (DREEM) ( Roff, 2005). In addition, we added questions addressing certain demographic and academic information. However, all data were obtained in anonymous manner, with participants only being asked to fill-in non-identifying academic and demographic data.

We obtained ethical approval on May 15, 2020 from the Community Medicine Department Technical and Ethical Review Board, Faculty of Medicine, University of Khartoum with the serial number: 5/2020. Selected students received a link to access the survey form. We clearly informed each participant about the objectives of the study and obtained a written consent.

### Data analysis

We analyzed data using Statistical Package for Social Sciences (SPSS) version 20. We reported mean item (all 50), subscales, and total scores and interpreted them as per McAleer and Roff’s (2001) guidelines ( McAleer and Roff, 2001). We calculated the percentage of those with positive, uncertain and negative responses for each item, and presented particularly low score items that need attention.

We used Student’s t test to compare mean scores between different groups (males and females, achievers and underachievers in addition to clinical and subclinical students) for each subscale. Moreover, we compared total and subscale scores for different age groups, residence types as well as the 7 batches included in the study and the 6 academic years to which they refer; using ANOVA. We considered a difference between groups statistically significant when the P value is less than 0.05.

## Results/Analysis

We contacted 405 students to participate in the study, 341 (84.2%) filled the questionnaire of whom 231 (67.7%) were females. Respondents’ age varied between 18 and 29 years with a mean of 21.77 (2.139). The participants’ residence was mainly family houses (67.2%) or university dormitories (26.7%) with only 6.2% specified other types of residence. Participants represented seven distinct academic batches of whom 99 (29%) belonged to academic phase1 (year 1-2), 99 (29%) belonged to academic phase2 (year 3-4) and 143 (42%) belonged to academic phase3 (year 5-6).

### DREEM scores and subscales

The mean DREEM score for the school from the overall sample was 104.48/200, reflecting that students perceived the academic environment as more positive than negative. Regarding DREEM subscales, 4 subscales were perceived as more positive; namely those pertaining to teaching, teachers, academic self-perception and social self-perception. On a negative note, the atmosphere was perceived as having many issues.

Looking at the results items wise as presented in *Table 1,* 17 out of 50 items had a mean score of ≤ 2 reflecting the need for special attention. No item scored more than 3.5. Mean scores of individual items are tabulated in *Table 2* along with percentages of agreement, disagreement, and uncertainty. Particularly high scoring DREEM questions were “I have good friends in this school”, “The teachers are knowledgeable”, and “I’m confident about passing this year” which had mean scores of 3.30, 3.22, and 2.80 respectively. In comparison, the lowest scoring items were “This school is well timetabled”, “There is a good support system for students who get stressed”, and “The enjoyment outweighs the stress of studying medicine” with mean scores of 1.30, 1.31, and 1.65 respectively.

**Table 1.** DREEM subscales and total score: Quantitative data obtained from 341 medical student respondents from the Faculty of Medicine- University of Khartoum in the year 2020

Subscales	Minimum	Maximum	Mean	Std. Deviation	Interpretation
**Students’ perception of teaching**	5	45	24.95	6.829	A more positive approach
**Students’ perception of teachers**	9	38	23.41	5.025	Moving in the right direction
**Students’ academic self-perception**	1	32	18.01	4.588	Feeling more on positive side
**Students’ perception of atmosphere**	2	44	23.30	6.802	Many issues which need changing
**Students’ social self-perception**	4	27	14.80	3.891	Not too bad
**Total DREEM score**	45	177	104.48	19.787	More positive than negative

**Table 2.** DREEM individual items: Quantitative data obtained from 341 medical student respondents from the Faculty of Medicine- University of Khartoum in the year 2020

DREEM Item	Agree/Strongly agree	Uncertain	Disagree/Strongly disagree	Mean
**I’m encouraged to participate in class**	**35.5%**	27%	**37.5%**	**1.95**
**The teaching helps to develop my confidence**	**45.2%**	22%	**32.8%**	2.19
**The teaching encourages me to be an active learner**	54.3%	17.3%	**28.4%**	2.33
**The teaching is well focused**	**47.8%**	24.9%	**27.3%**	2.21
**The teaching helps to develop my competence**	51.3%	22.9%	**25.8%**	2.33
**I’m clear about the learning objectives of the course**	54.3%	21.1%	**24.6%**	2.31
**The teaching is often stimulating**	**40.5%**	28.7%	**30.8%**	2.10
**The teaching time is put to good use**	**37%**	27.6%	**35.5%**	**2.00**
**The teaching is student centered**	**37.5%**	24%	**38.4%**	**1.97**
**Long term learning is emphasized over short term learning**	**49.9%**	29.9%	**20.2%**	2.34
**The teaching is too teacher centered**	53.4%	29%	17.6%	2.47
**The teaching overemphasizes factual learning**	**43.4%**	**42.5%**	14.1%	2.32
**The teachers are good at providing feedback to students**	**33.7%**	24.9%	**41.3%**	**1.89**
**The teachers have good communication skills with patients**	52.2%	**33.7%**	14.1%	2.43
**The teachers are knowledgeable**	89.4%	8.2%	2.3%	3.22
**The teachers gives clear examples**	60.4%	27.3%	12.3%	2.56
**The teachers are well prepared for their classes**	53.1%	29.6%	17.3%	2.45
**The teachers provide constructive criticism here**	**32.8%**	**36.1%**	**31.1%**	2.02
**The teachers ridicule the students**	**34.3%**	**40.8%**	**24.9%**	2.12
**The teachers get angry in class**	59.2%	19.9%	**20.8%**	2.50
**The teachers are authoritarian**	**46.3%**	**36.7%**	17%	2.35
**The teachers are patient with patients**	56.3%	**33.4%**	10.3%	2.57
**The students irritate the teachers**	68.6%	20.2%	11.1%	2.76
**I’m able to memorize all I need**	**25.8%**	21.7%	**52.5%**	**1.66**
**Much of what I have to learn seems relevant to a career in medicine**	68%	13.8%	18.2%	2.58
**I feel I’ m being well prepared for my profession**	**31.4%**	**33.1%**	**35.5%**	**1.91**
**Last year’ s work has been a good preparation for this year’ s**	**42.8%**	28.4%	**28.7%**	2.14
**My problem solving skills are being well developed here**	**48.1%**	22%	**29.9%**	2.20
**I’ m confident about passing this year**	71.6%	19.9%	8.5%	2.80
**I have learned a lot about empathy in my profession**	60.1%	24.9%	15%	2.54
**Learning strategies which worked well for me before continue to work for me now**	**43.4%**	**32.3%**	**24.3%**	2.18
**The atmosphere is relaxed during lectures**	**33.1%**	21.7%	**45.2%**	**1.82**
**I feel able to ask the questions I want**	**33.7%**	17.9	**48.4%**	**1.79**
**I feel comfortable in class socially**	55.7%	17%	**27.3%**	2.28
**There are opportunities for me to develop interpersonal skills**	60.4%	21.4%	18.2%	2.45
**The atmosphere is relaxed during seminars and tutorials**	**37.2%**	17.3%	**35.5%**	2.10
**The enjoyment outweighs the stress of studying medicine**	**32.3%**	16.1%	**51.6%**	**1.65**
**The atmosphere motivates me as a learner**	**37.5%**	16.7%	**45.7%**	**1.91**
**I am able to concentrate well**	**37.5%**	24%	**38.4%**	**1.94**
**The atmosphere is relaxed during the ward teaching**	**32%**	**36.1%**	**32%**	**1.93**
**This school is well timetabled**	**23.5%**	12.6%	**63.9%**	**1.30**
**I find the experience disappointing**	**29.6%**	**33.4%**	**37%**	**1.94**
**Cheating is a problem in this school**	**33.4%**	29.3%	**37.2%**	**1.93**
**I have good friends in this school**	89.7%	4.1%	6.2%	3.30
**There is a good support system for students who get stressed**	**19.9%**	15.8%	**64.2%**	**1.31**
**I am too tired to enjoy this course**	51.6%	18.8%	**29.6%**	2.30

***Flags: Less than 50% Agree/Strongly Agree; More than 30% Uncertain; More than 20% Disagree/Strongly Disagree. Mean less than 2.***

### Factors associated with DREEM subscales and total score

Test results as summarized in *Table 3,* show the significant difference we found between different students’ age groups, academic phases and batches in regards to their perception of atmosphere (p-value 0.023, 0.001, and 0.013 respectively), with students in phase 1 perceiving the atmosphere more positively than students in phase 2 and 3. In terms of batches however, students in batch 96 had the highest perception while those in batch 92 had the lowest perception. Moreover, regarding residence, we found a significant difference in students’ perception of teachers as well as total DREEM scores between different residence groups (p-value 0.000 and 0.030) with students residing in university dormitories having a more positive perception. In addition, there was a significant difference in students’ perception of teachers between different genders (p-value=0.000), with females appearing to perceive their learning environment more positively regarding teachers. Furthermore, considering academic achievement, test results showed no significant difference in students’ perception of educational environment in all DREEM subscales between achievers and underachievers.

**Table 3.** Factors associated with DREEM subscales and total score: Quantitative data obtained from 341 medical student respondents from the Faculty of Medicine- University of Khartoum in the year 2020

	Students’ perception of teaching	Students’ perception of teachers	Students’ academic self-perception	Students’ perception of atmosphere	Students’ social self-perception	Total DREEM score
**Gender**						
Female	25.42	24.17	18.01	23.13	14.75	105.48
Male	23.98	21.82	18.02	23.65	14.91	102.38
P value	.070	**.000**	.992	.510	.723	.177
**Age**						
P value	.315	.653	.951	**.023**	.574	.337
**Residence**						
Family house	24.25	23.26	17.77	23.14	14.82	103.24
University dormitories	27.26	23.98	18.58	24.15	14.88	108.86
Others	22.57	22.57	18.19	21.43	14.24	99.00
P value	**.000**	.379	.358	.207	.787	**.030**
**Academic phase**						
Phase 1	26.09	23.69	18.18	25.08	14.80	107.84
Phase 2	25.07	23.85	17.87	23.56	14.90	105.24
Phase 3	24.08	22.92	18.00	21.90	14.73	101.63
P value	.078	.297	.891	**.001**	.949	.050
**Batch number**						
96	27.23	23.96	18.89	25.98	15.66	111.72
95	25.06	23.44	17.54	24.27	14.02	104.33
94	25.89	24.48	17.48	23.34	14.79	105.98
93	24.00	23.02	18.37	23.84	15.05	104.28
92	24.64	23.11	16.86	21.25	13.56	99.42
91	24.49	22.84	19.11	21.53	15.00	102.96
90	23.27	22.87	17.62	22.73	15.27	101.75
P value	.099	.570	.167	**.013**	.165	.111
**Academic achievement**						
Achievers	25.31	23.37	17.98	23.47	14.66	104.80
Under-achievers	23.82	23.55	18.11	22.77	15.24	103.49
P value	.084	.775	.830	.416	.237	.603

**Flags: significant p values. Means for age groups were ignored due to the large number of age groups.**

## Discussion

The overall DREEM score was 104/200 indicating that perception was more positive than negative with a room for improvement. Higher overall DREEM scores were reported in earlier studies conducted in Sudan with mean scores of 122/200,132/200 respectively ( Ahmed *et al.,* 2018; Salih *et al.,* 2018). Regarding global scores, however, additional relatively higher scores were reported in UK and Spain with an overall DREEM score of 125/200,129/200 respectively ( Dunne, McAleer and Roff, 2006; Palomo-lópez *et al.,* 2018). While another study in India reported a lower overall DREEM score of 101/200. Having a wide range of disparity in DREEM scores nationally and globally suggests that geographical location plays a minor role in determining students’ perception of their educational environment and affirms the role of curriculum (universities that run teacher-centered, traditional curricula report similar low global scores compared to universities that run students-centered curricula).

### Students’ perception of teaching

In this subscale, students’ perception was viewed more positively than negatively. A previous study conducted in the same medical school reported similar perception in the same subscale ( Abdullahi, Elshazali and Shaheen, 2019), reflecting the efforts dedicated to maintaining the satisfaction of students and the attention needed to further nurture that satisfaction.

In this subscale, the three statements that scored 2 points or less were “I’ m encouraged to participate in class”, “the teaching is student-centered”, and “the teaching time is being put to good use”. All other items pertaining to perception of teaching had scores that indicated the presence of a room for improvement. We believe that the lack of student-centered teaching and encouragement for students’ participation is a result of the high number of students admitted in surplus of the faculty’ s capacity. The students might perceive teaching time as poorly used due to shorter time being spent in peer learning and the cancellation of scheduled sessions, particularly, clinical sessions where students spend a considerable time moving to and from the university’ s teaching hospital located in the outskirts of Khartoum.

### Students’ perception of teachers

Students’ perception regarding teachers was moving in the right direction; emphasizing teachers’ efficiency mainly attributed to the university’ s strict employment policy favoring highly qualified individuals. Findings in this subscale were similar to those of medical schools in Sudan, India, and Thailand ( Bhosale, 2015; Hongkan *et al.,* 2018; Salih *et al.,* 2018).

While the students perception of teachers’ knowledge was excellent and was rated as one of the highest scoring DREEM items in this institution, their feedback skills was the only item that scored less than 2 in this subscale. A similar area of concern has been noted in medical schools in UK, India, and Pakistan ( Dunne, McAleer and Roff, 2006; Kohli and Dhaliwal, 2013; Mushtaq *et al.,* 2017). The negative response to this item may reflect inappropriate feedback methods rather than a total absence. Ineffective feedback can pose educational barriers and undermine students’ confidence, especially when done in a judgmental or public manner. Thus, medical educators in this institution are encouraged to develop skills of giving timely, non-condescending feedback.

### Students’ academic self- perception

In this subscale, students were feeling more on the positive side. Similar results were reported in two separate studies conducted in Sudan ( Ahmed *et al.,* 2018; Salih *et al.,* 2018). A possible explanation is the not very well-prepared learning system-as shown by the SPL results- causing students to drift towards a self-dependent form of studying leading them to attribute their academic success to their own hard work.

Out of the eight items in this subscale, the two areas of concern pertained to student’ s ability of memorization and their preparedness for their profession. These observations -while not unique to our institution ( Kohli and Dhaliwal, 2013; Fahal, 2014; Bhosale, 2015)- raise special concerns seeing that over half (52.5%) of students reported not being able to memorize all they need and less than a third (31.4%) consider themselves well prepared for their profession. The academic overload can lead to the students particularly perceiving these two areas as deficient. Curricular modifications efforts should take into account the right methodology and content that doesn’t overload the students’ academic coping skills ( Bhosale, 2015).

### Students’ perception of atmosphere

Students’ perception regarding the atmosphere indicated many issues. Overcrowded lecture rooms can contribute negatively to the perception in this subscale. Findings in this subscale were consistent with those of a number of global studies ( Mayya and Roff, 2004; Enns *et al.,* 2016; Altemani and Merghani, 2017).

This subscale showed a number of weak areas with nine out of its twelve items scoring less than 2 as can be noted in Table 1. The lowest scoring item in this subscale -pertaining to the school’ s poor timetable- was as well the lowest scoring in the overall scale. This finding draws attention to students’ dissatisfaction with the current scheduling of weekly and annual timetable that leads to overlapping in academic years and eventually, leading to students spending a longer time in their undergraduate programs than is usually expected. The unexpected and unpredictable timetable leads to hindering students’ academic and personal post-graduation plans.

### Students’ social self-perception

In terms of students’ social self-perception, this institution was not too bad. The social atmosphere among peers in this particular institution has always been anecdotally praised. In the years 2016/2017, students in the same institution had a lower social self-perception ( Hamdan *et al.,* 2020). This improvement can provide incentives for continuous betterment of the social conditions in the faculty. Globally, a number of studies reported similar findings in this subscale ( Bhosale, 2015; Mushtaq *et al.,* 2017).

Items that scored less than 2 in this subscale pertained to boredom and the poor support system for students who get stressed. The problem of a poor support system was also reported by others ( Demirören *et al.,* 2008; Kohli and Dhaliwal, 2013; Altemani and Merghani, 2017). This institution has a mentoring program where faculty members are assigned to groups of student mentees. Improving reach of such programs and ensuring their execution with proper follow-up can alleviate stress and provide support for students ( Kohli and Dhaliwal, 2013). Medical students who get bored during their academic life are not an uncommon finding in medical schools ( Mayya and Roff, 2004; Kohli and Dhaliwal, 2013). An innovative curriculum that is more engaging to students can reduce students’ boredom ( Bhosale, 2015).

### Factors associated with DREEM subscales and total score

Although gender wise comparison of students’ perception across the DREEM subscales tended to follow a certain pattern with either females in some studies ( Brown, Williams and Lynch, 2011; Ugusman *et al.,* 2015) or males in others ( Salih *et al.,* 2018) having a more positive perception, our study showed no specific pattern. In terms of age and academic level, however, the results showed a pattern of decreasing mean scores as students progress in age and by default academic level or phase that is consistent with the findings of another study from India that assumed that students become more critical to the educational environment with time ( Walankar *et al.,* 2019).

In converse to a similar study conducted in the same medical school ( Abdullahi, Elshazali and Shaheen, 2019), our study results show a significant difference in total DREEM score in addition to students’ perception of teachers between different residence groups, with students residing in university dormitories having a better perception. This despite being inconsistent with the poor quality of living conditions in the university dormitories; can still be justified by the academically and socially supportive environment created between the residents of the university dormitories.

Similar studies conducted in Saudi Arabia ( Al-qahtani, 2015) and Malaysia ( Ugusman *et al.,* 2015) reported the same finding of our study; that no significant difference is detected in perception of educational environment between academic achievers and under-achievers. In spite of being inconsistent with the findings of several similar studies including a study conducted in a Sudanese medical school ( Abdullahi, Elshazali and Shaheen, 2019), our findings can yet be rationalized by the predominance of other factors rather than the academic achievement on their effect on the students’ perception of their educational environment.

### Limitations and strengths

One limitation of the study is that it relies merely on a quantitative base of evidence; making it unable to give a deep understanding of students’ perception and the origin of the weaknesses detected in different aspects of the educational environment. However, the study, through following a randomized method of participants’ selection; with consideration to gender groups and academic batches ratio, managed to deliver generalizable results those can be used as starting point for developmental plans and future studies.

## Conclusion

The study managed to draw a clear picture of how medical students at the University of Khartoum view their educational environment. Despite the general perception being more positive than negative, the study highlighted various areas needing special attention. In addition, variations reported in the perception of different demographic and academic groups define the need for further studies; especially those with a qualitative approach for a deeper understanding of the situation. Moreover, we believe that both faculty administration and students must work together to deliver tangible improvement in all aspects of educational environment.

## Take Home Messages

• The general perception was found to be more positive than negative. However, the study highlighted various areas for improvement.
• The answer to the main inquisition of our study is that no significant difference was detected in perception of educational environment between academic achievers and under-achievers.
• Mean DREEM scores showed no discrepancies in patterns of perception among different gender groups.
• Results showed a decremental pattern of students’ perception with increasing age and academic year.
• Surprisingly, this study revealed significantly higher DREEM scores for students living in dormitories compared to other residence-based groups.

## Notes On Contributors

**Duha Bushra Ahmed Mohammed** is a student at University of Khartoum, Faculty of Medicine, Khartoum state, Sudan. ORCiD: https://orcid.org/0000-0002-2891-0749

**Eilaf Abobaker Osman Yassen** is a student at University of Khartoum, Faculty of Medicine, Khartoum state, Sudan. ORCiD: https://orcid.org/0000-0002-8425-4264

**Mozan Azhary Hassan Mohamed** is a student at University of Khartoum, Faculty of Medicine, Khartoum state, Sudan. ORCiD: https://orcid.org/0000-0002-6166-6151

**Raghad Eltayeb Abdalla Eltayeb** is a student at University of Khartoum, Faculty of Medicine, Khartoum state, Sudan. ORCiD: https://orcid.org/0000-0003-1829-0563

**Rofida Salah Babiker Asmally** is a student at University of Khartoum, Faculty of Medicine, Khartoum state, Sudan. ORCiD: https://orcid.org/0000-0003-4913-0546

**Sara Abdellateef Mohammed Mohammed** is a student at University of Khartoum, Faculty of Medicine, Khartoum state, Sudan. ORCiD: https://orcid.org/0000-0002-5497-5837

**Sujood Fathi Elbashir Elhassan** is a student at University of Khartoum, Faculty of Medicine. ORCiD: https://orcid.org/0000-0002-4409-5916

**Gehad Abdelmonem Abdallah Ibrahim** is a graduate of University of Khartoum, Faculty of Medicine and a member of Sudan Medical Specialization Board, Khartoum state, Sudan.

**Note:** Contributions made by the listed authors were assigned on the base of allowing the first seven authors - those are third year medical students - to have a fair chance to participate substantially and in a peer-learning manner in all steps of scientific research conduction. Thus, the first seven authors worked as one students’ research team hence are all co-first authors and their names appear in alphabetical order. In addition, the last name appearing is the supervisor of this research team.
