# Supplementary material for: Educational environment and its relation to academic performance as perceived by medical students in University of Khartoum. A cross Sectional Study, Sudan, 2020
Source: MedEdPublish (2016). 2021 Jun 11;10:170. [Version 1] doi: 10.15694/mep.2021.000170.1 (PMC10939576; doi:10.15694/mep.2021.000170.1)
Supplement: Peer review history

# Reviewer report: Reviewer response for version 1

**DOI:** 10.21956/mep.19093.r27511

**Published:** 2021-07-15

**Authors:** Haipeng Xiao

**Affiliations:** 1 The First Affiliated Hospital, Sun Yat-sen University

This review has been migrated. The reviewer awarded 3 stars out of 5 The authors should first be congratulated on publishing this paper of considerable relevance in their local context, while all but one of them are still medical students!This is a cross-sectional study and the subject matter is the educational environment of the University of Khartoum in Sudan. The time-tested DREEM framework was used to investigate. One of the strengths of this study is the way the researchers went to great lengths for a sufficiently representative sample. Another glow of this paper is the conscientiousness of the authors by way of putting the results in perspective. As for areas of improvements, I tend to think that these findings (and the interpretation thereof) were relatively unsophisticated and granular. It would be much better, as the authors have already pointed out, to conduct a follow-up qualitative study for more in-depth explorations of why what did and did not work for whom.

Reviewer Expertise:

NA

No decision status is available

# Reviewer report: Reviewer response for version 1

**DOI:** 10.21956/mep.19093.r27512

**Published:** 2021-07-15

**Authors:** Barbara Jennings

**Affiliations:** 1 Norwich Medical School

This review has been migrated. The reviewer awarded 4 stars out of 5 I really enjoyed reading this paper about the use of the Dundee Ready Educational Environment Measurement (DREEM) inventory to evaluate learner experience of a six year undergraduate medical degree programme. I think it will be of interest to programme directors, managers and module leads. The noted changes in perception over time are interesting and I was reminded of the utility of the DREEM evaluation tool for capturing important information about the overall learning environment – all too often we focus our attention on fleeting operational issues and reductive subjective experiences when we audit our programmes. Checking in on the well-being of our learners, and how organisations are coping and developing, has never been more important as we cope with the upheaval of course expansions and the global pandemic. The introduction and discussion included some key citations that will be of interest to course faculty teams and leaders who are interested in evaluation methods.The methods section and results presentation were real strengths too. The authors outline appropriate governance for an educational research project and had carried out power calculations to plan appropriate data analysis.It was great to see that seven of the eight authors of this carefully written article were students.

Reviewer Expertise:

NA

No decision status is available
